# Geographical trends of PFAS in cod livers along the Norwegian coast

**DOI:** 10.1371/journal.pone.0177947

**Published:** 2017-05-22

**Authors:** Stig Valdersnes, Bente M. Nilsen, Joar F. Breivik, Asbjørn Borge, Amund Maage

**Affiliations:** 1 National Institute of Nutrition and Seafood Research (NIFES), Nordnes, Bergen, Norway; 2 Institute of Marine Research, Nordnes, Bergen, Norway; University of Siena, ITALY

## Abstract

The level of perfluorinated alkyl substances (PFAS) was determined in North East Arctic cod (*Gadus morhua*) liver samples from 15 Norwegian fjords and harbors. Five harbors in the eastern part of Norway, six harbors in the western part and four harbours in the northern part. A total of 200 samples were analyzed for 16 PFAS. Determination of PFAS were carried out by LC-MS/MS following sample clean up by solid phase extraction and ultracentrifugation. The predominating PFAS was PFOS, which was found to be higher than the level of quantification (1.5 μg kg^-1^ wet weight) in 72% of the samples. The highest level of PFOS found was 21.8 μg kg^-1^ wet weight in a sample from Kragerø in the eastern part of Norway. A significantly higher level of PFOS was found in the eastern fjords and harbors compared to fjords and harbors in the western and northern part of Norway. Within the northern fjords and harbors elevated PFOS levels were found in Narvik, which may indicate a local source there. Variations in PFOS of the cod livers thus reflect differences in levels of pollution between the areas.

## Introduction

Per- and polyfluorinated alkyl substances (PFAS) are anthropogenic compounds with unique properties due to the inertness of the carbon fluorine bond and the lack of water and oil solubility of the perfluorinated carbon chains [1]. These unique properties have led to widespread industrial use and inclusion in consumer applications in *e*.*g*. firefighting foams, products for stain and water repellency and lubricants. PFAS have shown both to bio accumulate and to be persistent in biota, although depuration of PFAS has been demonstrated from edible tissue of wild caught invertebrate species [2,3,4]. Two of the most studied and ubiquitous PFAS are perfluorooctane sulphonate (PFOS) and perfluorooctane carboxylic acid (PFOA). PFOS has been added to the Stockholm Conventions’ B-list, and all countries which have signed this treaty are thus obliged to limit production and use of this chemical [5]. Such limitations have been implemented in the European Union (EU), which prohibits production, import, export and sale of products containing more than 0.005 weight percent of PFOS and/or its precursor perfluorooctane sulfonyl fluoride (PFOS-F) [5]. Recently, the European Commission also included PFOS and its derivatives on the list of priority substances that must be monitored in EU water bodies and set an environmental quality standard (EQS) for PFOS and its derivatives of 9.1 μg kg^-1^ wet weight for fish [6]. In relation to food safety, no maximum levels have so far been set for the concentration of PFAS in fish or other foodstuffs placed on the market. In 2008, the European Food Safety Authority (EFSA) issued a scientific evaluation of PFOS and PFOA pointing out that there was a substantial lack of analytical data on levels in food items [7]. The limited data available, however, indicated that consumption of fish was one of the main routes of human exposure to PFOS. No convincing evidence for adverse effects of PFOS and PFOA in the general human population was found, although animal studies have shown hepatotoxic and carcinogenic effects as well as developmental and reproductive toxicity and neurotoxic effects, but the mechanisms involved are not well understood [7]. Based on the available data, EFSA established a tolerable daily intake (TDI) value of 150 ng kg^-1^ bodyweight for PFOS and 1.5 μg kg^-1^ bodyweight for PFOA. EFSA recommended more data to be collected on the levels of PFAS in food and people and this request has been implemented in European legislation [8,9].

A large number of studies on PFAS in a wide range of species have been performed, and in all species PFOS is the predominant PFAS detected [10,11]. In fish, elevated concentrations of PFOS (above 100 μg kg^-1^ wet weight) has been detected in liver, muscle and blood samples from various species and locations worldwide. Levels in fish are higher in liver and blood than in muscle, and higher in samples collected from more urbanized/industrialized regions. In Norway, data collected under the Oslo and Paris Commissions (OSPAR) Coordinated Environmental Monitoring Programme (CEMP) has shown that the median PFOS in cod liver from an urbanized location close to the capital Oslo city was significantly higher (up to 49 μg kg^-1^ w.w. in 2006) than other stations from more remote areas along the Norwegian coast [12]. In a recent study of cod from the Gulf of Gdansk, PFOS was also found to predominate in whole blood with a range from 6.1 to 52 pg mL^-1^ and a mean of 17 pg mL^-1^ [13].

Lean fish like Atlantic cod *(Gadus morhua)*, store energy as fat in the liver from which energy can be mobilized if needed. Fat soluble contaminants accumulate in the liver of such fish, and cod liver have been shown to contain high levels of fat soluble POPs like dioxins and dioxin like PCBs [14]. PFAS, however, bind to blood and liver proteins and accumulate in blood, liver, bile and kidney and most long chain PFAS are neither fat nor water soluble [3,15,16]. The bio accumulation factor (BAF) of PFAS spans orders of magnitudes, increases with longer perfluoralkyl carbon chain and sulfonates generally have higher BAF than carboxylates for equal chain lengths, although exposure concentrations typically are much higher than environmental concentrations [17,18,19,20].

The Atlantic cod is an important commercial species where both the muscle and the liver is consumed, the liver mainly to manufacture cod liver oil with high levels of nutritionally important omega-3 fatty acids. Cod is essentially a bottom dwelling fish, but can also remain pelagic during periods of its life span [21]. There are large stocks in the Barents Sea, the North Sea and also coastal stocks, living as a top predator feeding on a wide variety of prey species [22,23]. Distribution of this species is from the head of the fjords and out to the continental shelf slope (Eggakanten). Tagging experiments have shown that the coastal cod do not make large migrations [24]. It is still uncertain whether the coastal cod in the outer areas make major migrations. Due to the site specificity of this species, Norwegian coastal cod is suitable for the identification of potential point sources of manmade pollutants like PFAS as well as providing a baseline of PFAS provided by the coastal currents.

The purpose of this study was to establish the levels of PFAS in cod liver from fjords and harbors along the Norwegian coast and investigate differences and trends in relation to geography and biological factors.

## Materials and methods

### Sampling and sample preparation

**Fig 1:**
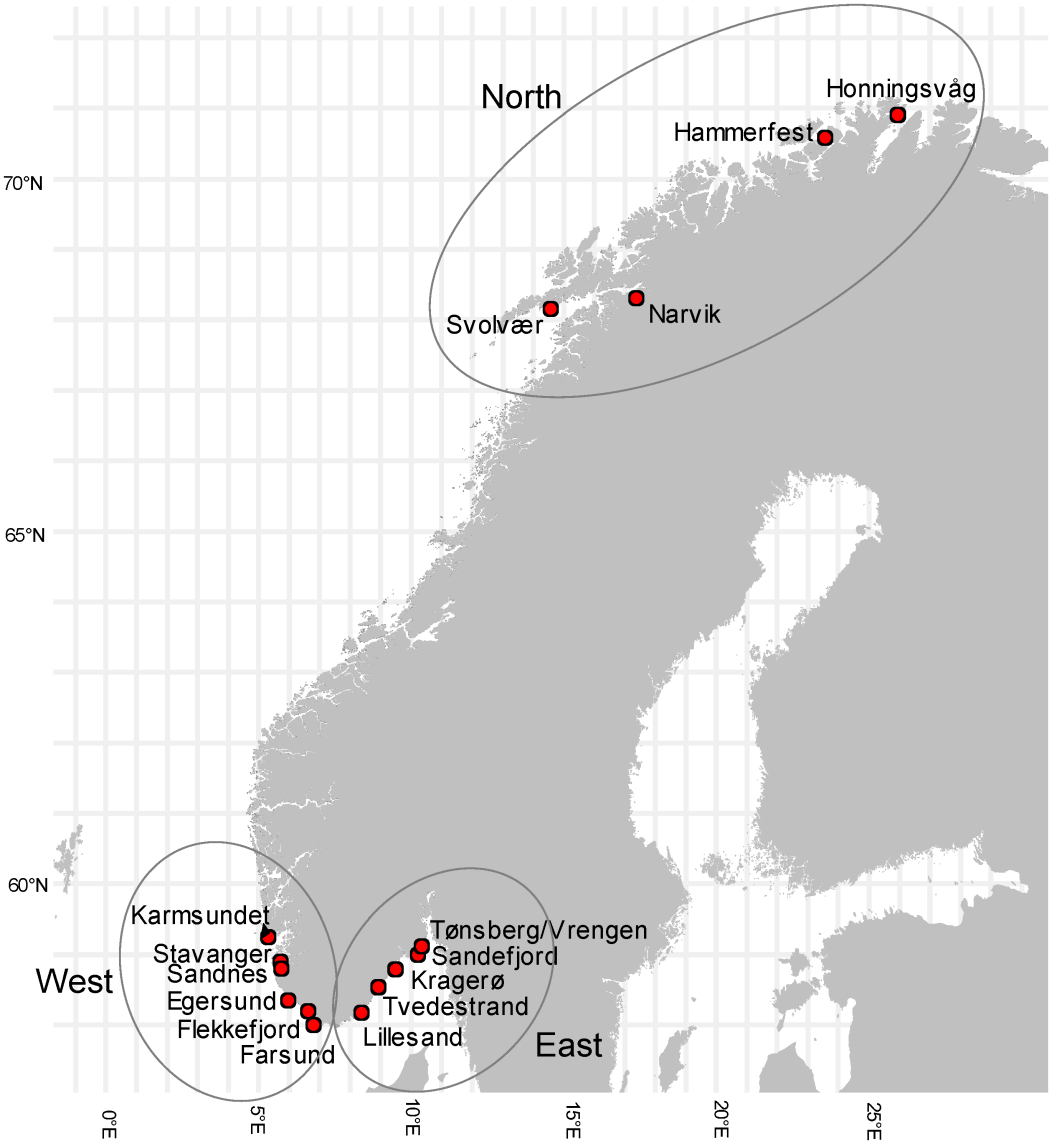
Map showing the 15 fjords and harbors where cod were sampled. The geographical areas North, West and East are indicated by circles.

A total of 200 cod from 15 fjords and harbors (Table 1, Fig 1) were collected by the Institute of Marine Research (IMR) or its reference fleet of fishermen, from 10th December 2008 to the 3rd November 2009 using a variety of fishing tools including nets, hand lines, fish pots and fish traps. The number of fish collected varied between 1 and 30 fish from each fjord/harbor. All fishermen involved in sampling were authorized for commercial fishing by the Norwegian Directorate of Fisheries and the fishing was carried out according to Norwegian laws regulating the catch, handling and euthanasia of wild caught fish. Total length and weight of the fish from Lillesand, Tvedestrand and four stations in Kragerø were determined on the sampling vessels before the liver of each fish was collected in a clean glass container with a tin plate lid lined with a PVC based B60 plastisol sealing gasket. Liver samples from these stations were frozen (-20°C) and sent to NIFES. Fish from the remaining sampling stations were frozen as single whole fish in plastic bags and sent to NIFES. After arrival at NIFES, the round fish were thawed and the length, weight and sex were determined. Sex was determined by visual inspection of the gonads. Otoliths were removed for age determination. The otoliths were broken, and the annual growth zones visible on the broken sections were counted using a binocular microscope with light transmitted from the side with a shadowed surface [25]. The livers were dissected out and the rest of the fish frozen for future analyses. Liver samples were weighed and homogenized before analysis and determination of fat [26]. A total of 200 liver samples from 40 stations were determined for PFAS.

**Table 1:** Overview of physical and biological parameters of fish from the areas North, West and East and each fjord/harbor within these areas. The number of fish and the mean (min-max) values for the physical parameters are shown for each area. Na = not analysed. Different superscript lower case letters (a, b, c, d) indicate significant differences between fjords/harbors (Kruskal-Wallis test; p<0.05). Different superscript capital letters (A, B) indicate significant differences between the areas North, West and East (Kruskal-Wallis test; p<0.05).

Fjord/Harbor/Area	N total	N male	N female	Liver weight, g	Liver fat, %	Condition factor	Liver somatic index	Whole fish weight, g	Whole fish length, cm	Age†
Honningsvåg	12	6	6	53.4 (21.6–86.1)^b^	35.9 (22.8–59.1) ^bc^	1.17 (0.96–1.32) ^a^	0.0435 (0.0236–0.0794) ^ad^	1240 (689–1938) ^a^	46.9 (39–56) ^a^	4.3 (3–6)
Hammerfest	20	11	9	63.8 (30.7–183.4) ^b^	35.3 (8.7–55.3) ^bc^	1.00 (0.83–1.23) ^bc^	0.0284 (0.0106–0.0579) ^bd^	2247 (809–3483) ^ab^	60.2 (43–75) ^bcd^	5.9 (4–8)
Narvik	9	4	5	56.7 (24.1–88.5) ^ab^	12.3 (3.5–33.7) ^b^	0.92 (0.76–1.10) ^bc^	0.0129 (0.0097–0.0204) ^c^	4609 (2260–7320) ^b^	78.2 (59–95) ^b^	8.2 (6–12)
Svolvær	26	10	16	185.1 (40.3–489.0) ^a^	58.7 (34.4–74.0) ^a^	1.15 (0.91–1.61) ^ac^	0.0543 (0.0193–0.1070) ^a^	3108 (1440–5940) ^bc^	63.4 (51–77) ^bc^	Na
North	67	31	36	108.1 (21.6–489.0)^A^	41.4 (3.5–74.0) ^A^	1.08 (0.76–1.61) ^A^	0.0391 (0.0097–0.1070) ^A^	2718 (689–7320) ^A^	61.5 (39–95) ^A^	5.9 (3–12) ^A^
Karmsundet	19	6	13	73.5 (28.7–135.7) ^ab^	43.7 (29.6–56.8) ^ac^	1.06 (0.88–1.28) ^ab^	0.0329 (0.0127–0.0926) ^ab^	2454 (680–4460) ^bcd^	60.5 (40–75) ^bcd^	4.6 (2–7)
Stavanger	11	3	8	39.9 (21.0–69.9) ^b^	32.1 (12.3–50.5) ^bc^	0.98 (0.75–1.08) ^ab^	0.0234 (0.0121–0.0438) ^bcd^	1755 (963–3360) ^ac^	55.8 (46–70) ^ac^	3.4 (2–5)
Sandnes	3	1	2	44.7 (34.8–61.7) ^ab^	39.0 (31.6–49.5) ^abc^	1.07 (1.01–1.17) ^ab^	0.0208 (0.0177–0.0243) ^abc^	2175 (1547–3009) ^ab^	58.3 (51–66) ^ab^	3.7 (2–5)
Egersund	13	10	3	43.8 (24.8–79.8) ^b^	36.0 (21.1–54.2) ^bc^	1.06 (0.89–1.21) ^ab^	0.0227 (0.0132–0.0377) ^bc^	2012 (1240–3060) ^ab^	57.2 (49–69) ^ac^	Na
Flekkefjord	17	2	15	53.6 (27.2–106.0) ^b^	33.0 (13.4–45.3) ^bc^	1.01 (0.85–1.19) ^ab^	0.0230 (0.0133–0.0370) ^bc^	2393 (880–3860) ^bcd^	61.1 (42–76) ^bcd^	Na
Farsund	23	6*	16*	46.7 (28.1–101.0) ^b^	39.3 (8.7–57.7) ^c^	1.04 (0.79–1.34) ^ab^	0.0311 (0.0140–0.0585) ^ab^	1574 (840–3260) ^ad^	52.6 (42–71) ^ad^	Na
West	86	28*	57*	52.6 (21.0–135.7) ^B^	37.6 (8.7–57.7) ^A^	1.04 (0.75–1.34) ^A^	0.0273 (0.0121–0.0926) ^B^	2041 (680–4460) ^B^	57.3 (40–76) ^B^	4.3 (2–7) ^B^
Lillesand	3	Na*		36.4 (28.4–52.1) ^ab^	31.4 (18.0–54.5) ^abc^	0.99 (0.88–1.05) ^ab^	0.0257 (0.0185–0.0319) ^abc^	1463 (890–1950) ^ab^	52.3 (44–57) ^ab^	Na
Tvedestrand	7	Na*		48.0 (28.9–80.1) ^ab^	35.3 (22.8–45.3) ^abc^	0.92 (0.77–1.26) ^b^	0.0257 (0.0186–0.0405) ^abc^	1839 (1350–2280) ^ab^	58.6 (54–65) ^ab^	Na
Kragerø	30	2*	6*	68.2 (31.7–185.7) ^ab^	32.4 (7.9–67.4) ^bc^	0.96 (0.77–1.15) ^b^	0.0254 (0.0135–0.0689) ^bc^	2895 (970–6860) ^bc^	65.7 (46–90) ^bc^	6.3 (5–9)
Sandefjord	1	1		33.5 ^ab^	44.6 ^abc^	1.11 ^ab^	0.0310 ^abc^	1080 ^ab^	46.0 ^ab^	2
Tønsberg/Vrengen	6	3	3	38.0 (27.0–62.6) ^b^	37.0 (4.8–52.7) ^abc^	1.04 (0.89–1.16) ^ab^	0.0266 (0.0143–0.0392) ^abc^	1573 (720–2540) ^ac^	52.3 (41–62) ^ac^	3.5 (2–5)
East	47	6*	9*	58.6 (27.0–185.7) ^B^	33.7 (4.8–67.4) ^A^	0.97 (0.77–1.26) ^B^	0.0257 (0.0135–0.0689) ^B^	2439 (720–6860) ^AB^	61.7 (41–90) ^AB^	4.7 (2–9) ^AB^

* Sex was determined for 22 out of 23 fish from Farsund and for 8 out of 30 fish from Kragerø. For the fish from Lillesand and Tvedestrand, sex was not determined. Consequently, sex was determined for 85 out of 86 fish from the area West and 15 out of 47 fish from the area East.

^†^Age was determined in 81 of 200 cod

### Chemicals

LiChrosolv grade methanol (Merck, Darmstadt, Germany) and ultrapure Milli Q water with electrical resistivity 18.2 MΩcm^-1^ at 25°C was used. Ammonium acetate (99.999% trace metal basis) was obtained from Sigma-Aldrich (St. Louis, MO, USA). Formic acid (98–100%) with p.a. quality (Merck, Darmstadt, Germany) and ammonia solution (25%) of Suprapur quality (Merck, Darmstadt, Germany) was used. Native PFAS (PFAC-MXB) and isotope labeled PFAS (MPFAC-MXA and FOSA-M) solutions were obtained from Wellington Laboratories (Guelph, ON, Canada).

### Extraction and analysis

Samples were analysed at NIFES. The extraction method was based on two published methods [27,28]. Liver samples were weighed (0.5 g) in a PP-tube and internal standard solution added. Methanol was added (4 mL) and the sample extracted for 60 minutes using ultrasound followed by centrifugation for 10 minutes at 4000 rpm. The supernatant was decanted into a plastic syringe equipped with a 0.45 μm nylon filter (Millipore, Billerica, MA). The filtered extract was diluted to 35 mL by adding Milli Q water. The diluted extract was decanted into a plastic tube to be placed on the ASPEC (Gilson, Middleton, WI) for cleanup. Oasis WAX cartridges (3 cc, 60 mg, 30 μm) (Waters, Millford, MA) were conditioned with 1% ammonium hydroxide in methanol (5 mL), followed by methanol (5 mL) and Milli Q water (5 mL). The diluted sample extract was then loaded into the cartridge before the cartridge was washed with 2% aqueous formic acid (5 mL). After drying, the analytes were eluted using 1% ammonium hydroxide in methanol (1 mL). The eluate was transferred to a 3K ultracentrifugation filter (Millipore, Billerica, MA) and centrifuged. The cleaned extracts were analyzed on an Acquity UPLC (Waters, Millford, MA) connected to a Quattro Premier MS/MS (Waters, Millford, MA). The UPLC was fitted with a BEH C18 column (100 mm length, i.d. 2.1 mm, particle size 1.8 μm) with a BEH C18 guard column (50 mm length, i.d. 2.1 mm, particle size 1.8 μm) to remove PFAS originating from the instrumental parts pre column. The LC mobile phases were 70:30 (v/v %) water:methanol as A and methanol as B, both with 2 mM ammonium acetate added. The flow rate was set at 0.35 mL min^-1^ with a run time of 7.5 minutes and a column temperature of 60°C. The gradient started at 100% A changing linearly to 100% B during 6 minutes. This was held for 1 minute before returning to initial conditions. The MS/MS was operated in ESI negative multiple reaction monitoring (MRM) mode. Capillary was set at 1 kV, source temperature was set at 120°C and desolvation temperature was set at 400°C. Cone and collision voltages were set individually for each compound. All samples of cod liver were analyzed for the following 16 perfluorinated analytes; perfluoro-1-butanesulfonate (PFBS), perfluoro-1-hexanesulfonate (PFHxS), perfluoro-1-octanesulfonate (PFOS), perfluoro-1-decanesulfonate (PFDS), perfluoro-1-octanesulfonamide (PFOSA), perfluoro-n-butanoic acid (PFBA), perfluoro-n-pentanoic acid (PFPeA), perfluoro-n-hexanoic acid (PFHxA), perfluoro-n-heptanoic acid (PFHpA), perfluoro-n-octanoic acid (PFOA), perfluoro-n-nonanoic acid (PFNA), perfluoro-n-decanoic acid (PFDA), perfluoro-n-undecanoic acid (PFUDA), perfluoro-n-dodecanoic acid (PFDoDA), perfluoro-n-tridecanoic acid (PFTrDA), Perfluoro-n-tetradecanoic acid (PFTeDA). The following isotope labeled analytes were used as internal standards for the analytes in parentheses; 1,2,3,4-^13^C_4_-PFBA (PFBA), 1,2-^13^C_2_-PFHxA (PFPeA, PFHxA), 1,2,3,4-^13^C_4_-PFOA (PFHpA, PFOA), 1,2,3,4,5-^13^C_5_-PFNA (PFNA), 1,2-^13^C_2_-PFDA (PFDA), 1,2-^13^C_2_-PFUdA (PFUdA), 1.2-^13^C_2_-PFDoDA (PFDoDA, PFTrDA, PFTeDA), ^18^O_2_-PFHxS (PFBS, PFHxS) and 1,2,3,4-^13^C_4_-PFOS (PFOS, PFDS, PFOSA).

### Quality assurance

The method was validated in order to determine important performance criteria such as precision, accuracy (trueness as recovery), measurement uncertainty, level of detection (LOD) and level of quantification (LOQ) [29]. LOD was determined at three times the signal to noise and LOQ was set equal to three times the LOD. Since there was no certified reference material available for PFAS in seafood at the time of validation, validation was carried out with relevant samples. Spiked samples were used to determine the recovery. LOQ in μg kg^-1^ for the different PFAS, with the average recovery in %, were as follows: PFBS (4.5, 54%), PFHxS (0.9, 89%), PFOS (1.5, 91%), PFDS (0.9, 65%), PFOSA (2.7, 49%), PFBA (3, 96%), PFPeA (3, 99%), PFHxA (1.5, 94%), PFHpA (3, 78%), PFOA (1.8, 94%), PFNA (1.5, 89%), PFDA (0.9, 96%), PFUdA (1.5, 94%), PFDoDA (2.4, 93%), PFTrDA (2.4, 78%), PFTeDA (2.4, 56%). The method has participated regularly in proficiency tests since 2009 with z-scores < |2| for analytes abvove LOQ.

Samples were extracted and analyzed in batches of 16. In addition, four control samples were included in every run to monitor the quality of the method giving a total of 20 samples in each run. Control samples in each series consisted of one procedural blank, one matrix blank, one spiked matrix blank for recovery calculations and one sample which contained quantifiable amounts of several PFAS. Fish sample from the Quasimeme 2009 proficiency test for PFAS was used as the control sample with quantifiable PFAS concentration. PFAS were not present in either the procedural, instrument or matrix blank above the level of quantification (LOQ).

### Data analysis and statistical analysis

Unscrambler X 10.4 (64-bit) (CAMO Software AS, 2016) was used for principal component analysis (PCA) and STATISTICA 12 (StatSoft, Inc, 2013) was applied for all other statistical analyses and graphs. The map was made using Mapinfo 10.0.1 (Pitney Bowes Software Inc., USA). In the calculations, PFOS concentrations below the limit of quantification was assumed to be equal to the limit of quantification (upperbound concentrations, UB). Fulton’s condition factor (K) is defined as K = 100(W L^-3^), where W is the whole body weight in grams and L is the total length in centimeter [30]. The liver somatic index or hepatosomatic index (HSI) is defined as the ratio of liver weight to body weight of an animal [30,31].

PCA was performed with the variable PFOS (UB), fish length, fish weight, fish age, liver weight, liver fat, condition factor and liver somatic index. The grouping variable area (N = North, W = West and E = East) was used in the score plot. The area North consisted of the sites Honningsvåg, Hammerfest, Narvik and Svolvær (67 samples). The area West included Karmsundet, Stavanger, Sandnes, Egersund, Flekkefjord and Farsund (86 samples). Lillesand, Tvedestrand, Kragerø, Sandefjord and Tønsberg/Vrengen made up the area East (47 samples). Lindesnes, the southernmost point in Norway was used as a dividing point between East and West.

For the comparison of physical parameters between the different geographical areas (North, West and East) and between the different fjords/harbors Kruskal-Wallis test was used. For the comparison of PFOS (UB) concentrations between the different geographical areas (North, West and East) and between the four different fjords within the area North (Honningsvåg, Hammerfest, Narvik and Svolvær), one-way analysis of variance (ANOVA) followed by Tukey multiple comparison tests was used. Since the PFOS-concentrations were skewed (skewness 3.2, kurtosis 14.4) and showed heteroscedasticity with increasing variation with increasing mean values, logarithmic transformation was used to transform the data before statistical analysis. When comparing PFOS concentrations between all the 15 different fjords/harbors, log transformation did not remove the problem with heteroscedasticity, and Kruskal-Wallis test was therefore chosen for this analysis. A significance level of 0.05 and individual as replicate unit was used in all analyses.

## Results

### Physical parameters

The 200 cod livers analyzed weighed between 21 g and 489 g with a mean of 73 g and a standard deviation (SD) of 72 g and had a fat content between 3.5% and 74% with a mean of 38% and a SD of 16%. The fish weight varied from 680 g to 7320 g with a mean of 2361 g and a SD of 1254 g, and fish lengths varied between 39 cm and 95 cm with a mean of 60 cm and a SD of 11 cm. Age was determined in 81 of the 200 cods and varied from 2 to 12 years, with a mean of 5 years and a SD of 2 years. Of the 167 fish where sex was determined, 61% were females and 39% males. Results overview per fjord/harbor and area (North, West, East) are shown in Table 1.

Cod from the area North were on average significantly larger (p < 0.05) and older (p < 0.01) than fish from the area West, but not East (Table 1). Area North also showed higher liver weight (p < 0.05) and liver somatic index (p < 0.01) than fish from the areas East and West. Also fish from the area East were on average larger and older than fish from the area West, but these differences were not found to be statistically significant. Fish from the area East had lower condition factor (p < 0.01) than fish from both the areas West and North. The average liver fat was highest in fish from the area North and lowest in fish from the area East, but these differences were not found to be statistically significant.

When comparing fish from the different fjords and harbors, no significant differences were found in average size, age, liver weight, fat in liver, liver somatic index or condition factor between fish from individual fjords and harbors within the areas East and West. Within the area North, however, cod from Narvik were on average significantly larger (p < 0.0001) and had a lower condition factor (p < 0.005) than fish from Honningsvåg, a lower fat content in liver (p < 0.0001) than fish from Svolvær and a lower liver somatic index (p < 0.05) than fish from all the three other harbors in this area.

### PFAS

PFOS was the major PFAS found in the cod livers. Out of the 200 cod livers analyzed, 144 livers (72%) had a PFOS concentration above the LOQ of the method (1.5 μg kg^-1^ w.w.) (Table 2). The PFOS-concentrations were, however, rather low in most of the fish, and only eight fish (four from Kragerø, one fram Tvedestrand, Lillesand, Farsund, and Narvik) had PFOS concentrations in liver at or above the EQS value of 9.1 μg kg^-1^ w.w. The highest PFOS concentration (21.8 μg kg^-1^ w.w.) was found in a cod liver from Kragerø in the eastern part of Norway. For the other PFAS determined, concentrations above the LOQ of the method were found for PFUdA in 35 samples (mean±SD) (2.3±0.7 μg kg^-1^ w.w.), for PFTrDA in 20 samples (3.8±1.6 μg kg^-1^ w.w.) and for PFDA in 11 samples (1.3±0.8 μg kg^-1^ w.w.). PFOSA (4.0±2.0 μg kg^-1^ w.w.), PFDoDA (2.9±0.3 μg kg^-1^ w.w.), PFOA (2.6±0.4 μg kg^-1^ w.w.) and PFNA (2.4 μg kg^-1^) were found in concentrations above LOQ in four, three, two and one sample, respectively. PFBS, PFHxS, PFDS, PFBA, PFPeA, PFHxA, PFHpA, PFTeDA were not found in concentrations above the LOQ in any of the samples.

**Table 2:** Concentration of PFAS (μg kg^-1^) in liver of cod from the areas North, West and East and each fjord/harbor within these areas. The mean (min-max) values and the number of samples (N) with values above LOQ are shown for each area.* Different superscript lower case letters (a, b, c, d) indicate significant differences between fjords/harbors (ANOVA (North) and Kruskal-Wallis test (West & East); p<0.05). Different superscript capital letters (A, B) indicate significant differences in PFOS concentrations between the areas North, West and East (ANOVA; p<0.0001). Dates of capture of fish from the different areas are shown in a separate column.

Harbor	PFHxS	PFOS	PFOSA	PFOA	PFNA	PFDA	PFUdA	PFDoDA	PFTrDA	Dates of capture
Honningsvåg	< 0.9	1.9 (1.8–2.0) ^d^ N = 3	< 2.7	< 1.8	< 1.5	< 0.9	< 1.5	< 2.4	< 2.4	09.02.-20.04.09
Hammerfest	< 0.9	2.4 (1.5–3.7) ^d^ N = 10	< 2.7	< 1.8	< 1.5	< 0.9	1.7 N = 1	< 2.4	< 2.4	27.01–06.06.09
Narvik	< 0.9	4.4 (1.9–9.4) ^abc^ N = 9	< 2.7	< 1.8	< 1.5	0.9 N = 1	2.0 (1.9–2.1) N = 2	< 2.4	< 2.4	18.-21.05.09
Svolvær	2.3 (1.5–3.0) N = 2	3.0 (1.5–5.1) ^d^ N = 9	< 2.7	< 1.8	< 1.5	< 0.9	1.6 N = 1	< 2.4	< 2.4	10.12.08–30.03.09
North	2.3 (1.5–3.0) N = 2	3.1 (1.5–9.4)^A^ N = 31	< 2.7	< 1.8	< 1.5	0.9 N = 1	1.8 (1.6–2.1) N = 4	< 2.4	< 2.4	10.12.08–06.06.09
Karmsundet	< 0.9	2.2 (1.5–3.4) ^cd^ N = 12	< 2.7	< 1.8	< 1.5	< 0.9	< 1.5	< 2.4	< 2.4	13.-25.05.09
Stavanger	< 0.9	2.7 (1.5–4.2) ^bd^ N = 8	< 2.7	< 1.8	< 1.5	< 0.9	1.6 (1.6–1.6) N = 2	2.6 N = 1	3.9 (3.4–4.3) N = 2	21.04.-11.05.09
Sandnes	< 0.9	2.0 (1.9–2.1) ^ad^ N = 2	< 2.7	< 1.8	< 1.5	< 0.9	< 1.5	< 2.4	2.8 N = 1	05.-11.05.09
Egersund	< 0.9	1.8 (1.5–2.2) ^d^ N = 8	< 2.7	2.6 (2.3–2.9) N = 2	< 1.5	< 0.9	< 1.5	< 2.4	2.4 N = 1	02–04.05.09
Flekkefjord	< 0.9	2.8 (1.5–4.3) ^ad^ N = 16	< 2.7	< 1.8	< 1.5	< 0.9	2.4 (1.6–3.7) N = 8	3.1 (3.0–3.1) N = 2	4.8 (2.8–8.8) N = 8	05.05.09
Farsund	< 0.9	3.3 (1.5–15.6) ^ad^ N = 21	2.9 N = 1	< 1.8	< 1.5	2.7 N = 1	2.5 (1.5–3.6) N = 4	< 2.4	3.0 (2.5–3.5) N = 2	21–24.05.09
West	< 0.9	2.7 (1.5–15.6) ^A^ N = 67	2.9 N = 1	2.6 (2.3–2.9) N = 2	< 1.5	2.7 N = 1	2.3 (1.5–3.7) N = 14	2.9 (2.6–3.1) N = 3	4.1 (2.4–8.8) N = 12	21.04.-25.05.09
Lillesand	< 0.9	6.2 (2.7–11.7) ^ad^ N = 3	< 2.7	< 1.8	< 1.5	< 0.9	< 1.5	< 2.4	< 2.4	29.04.09
Tvedestrand	< 0.9	7.0 (3.1–9.3) ^ad^ N = 6	< 2.7	< 1.8	< 1.5	< 0.9	< 1.5	< 2.4	< 2.4	28.04.09
Kragerø	< 0.9	5.8 (1.8–21.8) ^a^ N = 30	3.1 (2.7–3.4) N = 2	< 1.8	2.4 N = 1	1.4 (3.1–7.0) N = 7	2.5 (1.6–3.2) N = 14	< 2.4	3.2 (2.8–3.5) N = 5	30.04–23.07.09
Sandefjord	< 0.9	2.3 N = 1 ^ad^	< 2.7	< 1.8	< 1.5	< 0.9	< 1.5	< 2.4	< 2.4	06.-14.05.09
Tønsberg/Vrengen	< 0.9	5.7 (4.2–6.8) ^ab^ N = 6	7.0 N = 1	< 1.8	< 1.5	0.95 (0.9–1) N = 2	1.9 (1.7–2.0) N = 3	< 2.4	2.4 N = 1	30.06–03.11.09
East	< 0.9	5.9 (1.8–21.8) ^B^ N = 46	4.4 (2.7–7.0) N = 3	< 1.8	2.4 N = 1	1.3 (0.9–3.1) N = 9	2.4 (1.6–3.2) N = 3	< 2.4	3.1 (2.4–3.5) N = 6	28.04.-03.11.09

* PFBS, PFDS, PFBA, PFPeA, PFHxA, PFHpA, PFTeDA were below LOQ (4.5, 0.9, 3, 3, 1.5, 3, 2.4, 24, 24 μg kg^-1^ w.w., respectively) in all samples. LOQ for PFHxS, PFOS, PFOSA, PFOA, PFDA, PFUdA, PFDoDA, PFTrDA were 0.9, 1.5, 2.7, 1.8, 0.9, 1.5, 2.4, 2.4 μg kg^-1^ w.w., respectively.

**Fig 2:**
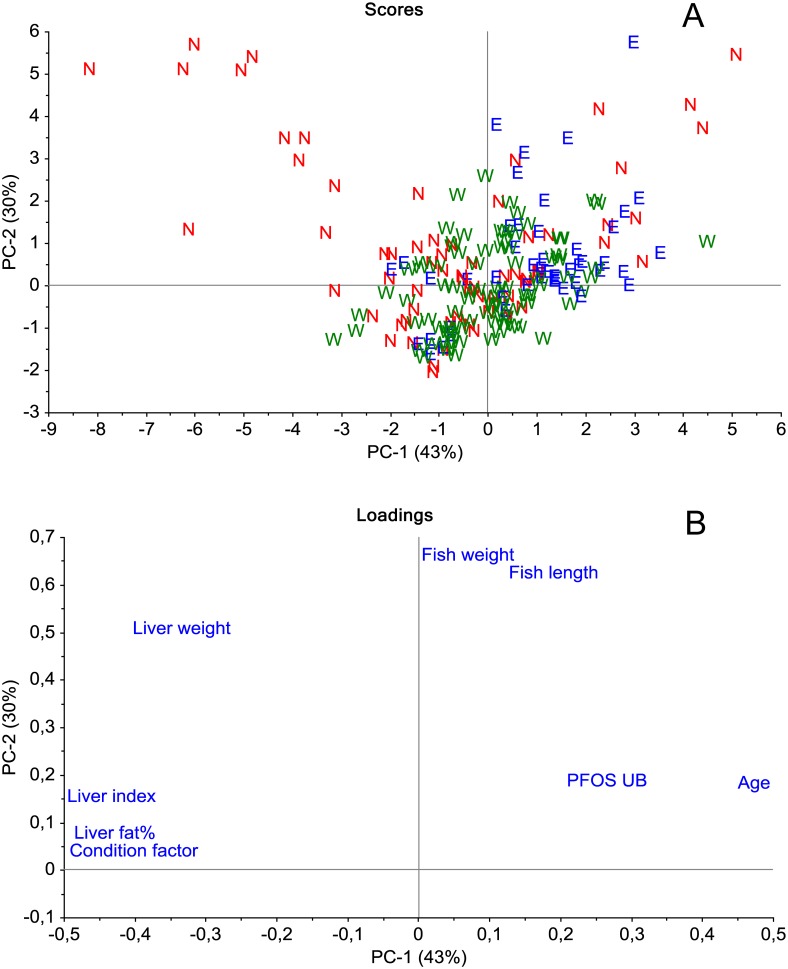
Score (A) and loading (B) plots of the PCA analysis on samples containing PFAS from different geographical areas (N = North, E = East and W = West) along the coast of Norway. The first two principal components explained 43% and 30% of the variance in the dataset.

The PCA score plot (Fig 2A) showed a partial separation of the data according to the different geographical areas, and the separation was mainly along the axis described by PFOS concentration, age and liver fat, liver somatic index and condition factor. The score plot also showed that cod from the area East generally had lower liver fat, liver somatic index and condition factor and higher PFOS concentrations, whereas cod from the area North had higher liver fat, liver somatic index and condition factor and lower PFOS concentrations. The PCA loading plot (Fig 2B) showed that the PFOS concentration was clearly negatively correlated with liver fat, liver somatic index and condition factor of the fish, positively correlated with age and only very weakly correlated with fish length, fish weight and liver weight.

**Fig 3:**
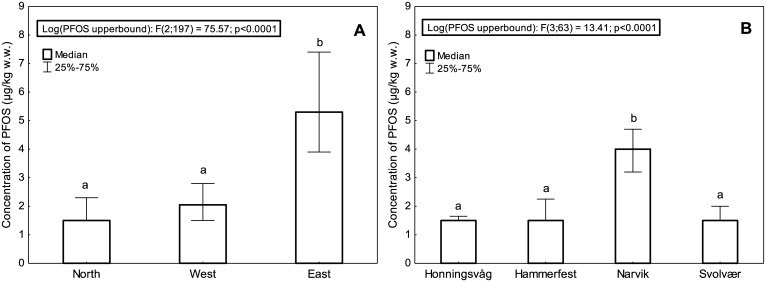
Median concentrations of PFOS in (A) different geographical areas and (B) different fjords within the area North. Upperbound PFOS concentrations were used and medians and 25% and 75% percentiles are shown. Results from one-way ANOVA with log transformed PFOS concentrations are shown. Different letters above the plots denote significant differences (p < 0.0001).

Comparison of the geographical areas using one-way ANOVA followed by Tukey and log-transformed PFOS concentrations, showed that liver from cod caught in the area East had significantly higher levels of PFOS (p < 0.0001) compared to liver from cod caught in the areas West and North (Fig 3A). No significant difference was found between the PFOS concentration in liver of cod from the area North and the area West. Within the area North there were, however, a significant difference between the individual harbors, in that the concentration of PFOS in cod liver from Narvik was significantly higher (p < 0.05) than the other harbors within that area (Fig 3B). Within the areas East and West, no significant differences in liver PFOS concentrations between the individual harbors were detected.

## Discussion

The oldest and largest fish (weight and length) was generally found to originate from the area North, although no significant difference were found compared to East (Table 1). The fish from area North also had the largest livers with the highest liver somatic index, whereas the condition factor of the fish from area East was significantly lower than for the other areas. The differences found may be influenced by the sampling and seasonal variations. Although Atlantic cod in Norwegian coastal fjords feed throughout the year, liver weights have been found to decline in the winter despite no apparent change in food intake [22,32,33,34]. However, this does not seem to be the case here since the samples in the area North were collected mainly in the winter and spring, whereas the other areas were collected in the spring and summer (Table 2), and the liver weight and somatic index was still higher in area North. The variation may also be influenced by other causes such as e.g. differential fishing pressure between areas, so caution must thus be used when interpreting these differences. Condition factor and liver somatic index are good stress indicators in fish, and changes or differences in these factors may reflect environmental changes and stress such as e.g. feed intake, metabolic rate and environmental pollutants [30,31]. Whereas the former is an indicator of the energy storage in the whole fish and changes in the condition factor can reflect both long and short term changes in environmental conditions, the latter is considered one of the most sensitive growth indicators responding more rapidly to environmental changes [35]. The lower condition factor of the fish from the area East may thus indicate increased environmental stress for the cods here compared to the other areas. The increased liver somatic index in the area North may also be an indication of a different environment, but it may also just be an effect of season and sampling differences. Nevertheless, as can be seen in Table 1, the harbor Svolvær was the main driver for the elevated liver weight and liver somatic index in the area North, and the harbor Narvik was the main driver for the larger size of fish from this area. From Table 1 it is also apparent that Tvedestrand and Kragerø are the main drivers of the lower condition factor in the area East.

The loading plot (Fig 2B) showed strong correlations between fish weight and fish length as expected. Age did not seem to be well correlated with length and weight of the fish, but the weak correlation could be due to rather few values in the PCA, since age was determined only in 81 of the 200 cods. Liver weight did not seem to correlate well with any of the other variables. Liver weight and liver fat percent did not correlate well with fish length or fish weight probably because the liver is used for energy storage and the amount of fat stored in the liver depends on the current energy intake of the cod and i.e. seasonal and temporal variations exists. A positive correlation between liver fat percent and liver somatic index can be seen from the loading plot and this is because energy is stored as fat in the liver. A positive association between condition factor and liver fat and liver somatic index was also evident from the loading plot. This can be explained by cod with high condition factor having high energy intake and growth of tissues, and that the storage of energy takes place in both muscle and livers [36,37].

The loading plot also showed that PFOS was weakly positively correlated with fish weight, length and age probably due to the bioaccumulation of this long chain PFAS [38]. On the other hand, PFOS was negatively correlated with condition factor and liver somatic index. Studies indicate that PFAS, such as PFOS, associates more strongly with protein rich compartments, such as the liver [39]. Associations between liver parameters such as liver weight, liver somatic index and liver fat percent and PFOS could therefore be expected. Hepatomegaly has previously been demonstrated in rats following exposure to mg kg^-1^ per day of PFOS and PFOA [40]. However, the levels of PFOS exposure to the wild cods in this investigation has probably been much lower than what Cui et. al. used, which could explain the weak impact of PFOS on the liver somatic index in this investigation. Nevertheless, the strong negative association between liver fat/liver somatic index and PFOS may be explained by the increased stress following the increased load of PFOS in the liver, or it may simply be a concentration effect due to the depletion of fat in the liver and a relative retention of PFOS causing an elevated level of PFOS in livers containing less fat. Although no difference in fat content was found between areas, the harbor Narvik which was a main driver for the elevated PFOS level found in the area North, had a low liver fat percent compared to all the other fjords/harbors although the difference was only significant for Svolvær, Karmsundet and Farsund. The lack of association between liver weight and PFOS in the PCA loading plot supports that the increased PFOS in low fatty livers is a result of a concentration effect.

**Fig 4:**
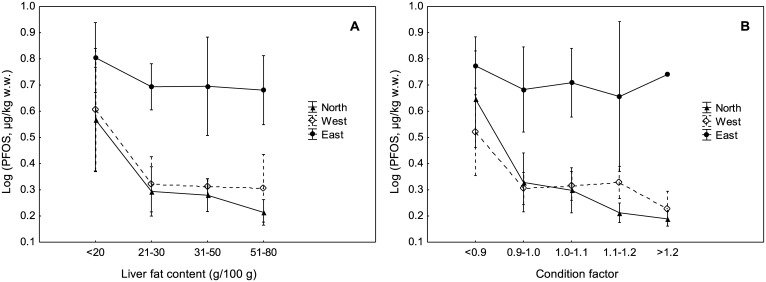
Plot of log-transformed upperbound PFOS concentrations against (A) liver fat classes and (B) condition factor classes for the three different geographical areas. Means and 95% confidence intervals are shown.

Further, the results showed that PFOS was generally higher in the eastern part of Norway (Fig 3A). Although differences in biological parameters were found between areas, such as a significantly lower condition factor for fish from the area East, when the PFOS concentrations were plotted against liver fat or condition factor for the three different geographical areas separately (Fig 4), the PFOS concentration was always higher in the area East than in the two other areas. Similarly, when the PFOS concentrations were plotted against fish length, fish weight and liver weight for each area separately, the PFOS concentration was always highest in the area East (results not shown). This indicates that the biological factors investigated in this study alone do not explain why the PFOS concentration was highest in the area East and lowest in the area North, and differences in PFOS levels between areas are influenced by geography.

There were also differences between the two fjords/harbors Narvik and Svolvær in Nordland County (Fig 3B), which could indicate local pollution variations between these harbors, with Narvik harbor affected. The harbor of Narvik has previously been identified as polluted by both tributyltin and polychlorinated biphenyls (PCBs), and due to the specific congener profile of the PCBs in Narvik it was concluded that a local source was likely [41]. PFAS were not part of this previous investigation, but our results indicate that PFAS pollution may also be part of the local contamination profile in Narvik. However, the cod from Narvik had a lower liver fat and a lower condition factor than most other cod from the three other harbors in the area North, which may also be part of the explanation why cod from this harbor had significantly higher PFOS concentrations in the liver.

Regarding food safety, it should be noted, that cod liver is a minor product for human diets, with little direct consumption. In a special traditional meal, “Mølje”, cod liver is eaten together with cod roe and cod fillet. It would, however, be rare to have more than 50–100 grams of cod liver in a meal and even with the highest single measurement in our study, 100 grams would give about 2 μg PFOS, which would be about 22% of the TDI for a 60 kg person [7]. As regards cod liver oil used as food supplements for human consumption, such oils are usually purified to remove contaminants and the levels found in cod liver may thus not be directly transferable to such products.

## Conclusion

The levels of PFAS in cod liver along the Norwegian coast were low. The dominant PFAS was PFOS, which was quantified in 72% of the livers and the highest concentration found was 21.8 μg kg^-1^ wet weight. We found geographical differences in the levels of PFOS, with the highest concentration in the area East, compared to North and West. It is likely that this difference is due to higher population density in the area East and its closeness to urbanized and industrialized regions in the Baltic and Northern Europe [12,13]. No significant differences were found between individual harbors within in the areas East and West, but within the area North we found higher levels in Narvik harbor. This should be further investigated to elucidate the possible local sources [41]. We also found geographical differences in the biological parameters of the cod collected within different areas. North had significantly higher liver weight and liver somatic index than the other areas and were significantly larger and older compared to area West, but not different from area East. Nevertheless, the results showed that the differences in the investigated biological parameters alone could not explain the variation in PFOS concentration between the areas. It is conceivable that both geographical and biological factors contribute to variations in PFOS levels, but further investigations are needed in order to evaluate the relative contribution of these factors in specific areas.

## Supporting information

S1 Table PFAS results. (XLSX)
